# The Effects of Electroacupuncture as an Adjunct Therapy on Poststroke Aphasia: A Systematic Review and Meta-Analysis

**DOI:** 10.1155/2022/1271205

**Published:** 2022-08-05

**Authors:** Yao Shi, Caixia Hu, Shuhua Li, Tianhua Huang, Xingsheng Chen, Guifu Li, Xiaohui Qin

**Affiliations:** ^1^Department of Neurology, The Second Affiliated Hospital of Guangzhou University of Chinese Medicine (Guangdong Provincial Hospital of Chinese Medicine) Guangzhou, Guangzhou, Guangdong, China; ^2^Department of Neurosurgery, The Second Affiliated Hospital of Guangzhou University of Chinese Medicine (Guangdong Provincial Hospital of Chinese Medicine) Guangzhou, Guangzhou, Guangdong, China

## Abstract

**Background:**

To systematically collate, appraise, and synthesize evidence of electroacupuncture (EA) as an adjunct therapy for poststroke aphasia (PSA) from randomized controlled trials (RCTs) through a systematic review and meta-analysis.

**Methods:**

An electronic search was conducted in eight databases to identify RCTs evaluating EA adjuvant therapy versus speech and language therapy (SLT). Methodological quality of the included trails was assessed by the Cochrane risk of bias. The software Review Manager 5.4 was used for data analysis.

**Results:**

Nineteen RCTs enrolling a total of 1263 subjects were identified. The use of EA combined with speech and language therapy (SLT) showed significant improvements in effective rate (RR 1.31, 95% CI [1.21, 1.41]), oral expression score (SMD 1.34, 95% CI [1.13, 1.56]), comprehension score (SMD 1.95, 95% CI [0.88, 3.03]), repetition score (SMD 1.84, 95% CI [0.75, 2.93]), naming score (SMD 1.97, 95% CI [0.81, 3.13]), and reading score (SMD 1.55, 95% CI [1.07, 2.04]) compared to the use of SLT alone.

**Conclusions:**

The pooled data indicate that EA combined with SLT for the treatment of PSA may improve clinical effectiveness, compared with SLT alone. Future high quality RCTs with large samples are still needed to confirm and expand our findings.

## 1. Introduction

Stroke is the most common cause of mortality and morbidity worldwide. Globally, more than ten million new cases of stroke are reported each year and at least one third of the affected individuals live with aphasia [1, 2]. Economic and social consequences are highly relevant because poststroke aphasia (PSA) has a serious negative impact on patients' activities of daily living [3]. Furthermore, the impact of PSA on functional communication, everyday activities, and social abilities of patients is dramatic and is, therefore, essential for the effective management and rehabilitation of aphasia [4]. Clinically, speech and language therapy (SLT) remain the gold standard for the treatment of PSA [5]. However, the clinical efficacy of this therapy still cannot meet patients' expectations [5]. In this situation, some patients choose complementary and alternative therapies to treat PSA in an effort to improve their quality of life.

In China, acupuncture is a widely used clinical rehabilitation technique, which is also recommended as an alternative treatment option for poststroke rehabilitation by the Ottawa Panel clinical practice guidelines [6]. As sources of the highest level of evidence for evidence-based medicine, previous systematic reviews/meta-analyses [7–13] have almost all revealed the benefits of acupuncture on PSA. As an extended technique of acupuncture, electroacupuncture (EA) has both the effects of traditional acupuncture and the functions of modern electrotherapy [14]. A recently published network meta-analysis [15] concluded that the efficacy of EA combined with SLT for PSA was superior to that of SLT alone. In addition, a systematic review [16] conducted in Korea concluded that EA could be considered as an adjunctive therapy for PSA. Nevertheless, the relative effect of EA on PSA could not be assessed because quantitative synthesis was not performed. A preliminary literature search identified a growing number of randomized controlled trials (RCTs) on the effects of EA for PSA, whereas, controversial efficacy was reported. Thus, to systematically collate, evaluate, and synthesize current evidence, we conducted this study.

## 2. Methods

This meta-analysis was carried out following the guidelines of Cochrane handbook [17] and updated PRISMA checklists [18]. The protocol was registered in the PROSPERO database (no. CRD42021254369).

### 2.1. Literature Search and Selection

PubMed, the Cochrane Library, Web of Science, Embase, CNKI, Wanfang, VIP, and CBM were systematically searched from database establishment to June 2022. Stroke, aphasia, electroacupuncture, and randomized controlled trials were applied as search keywords. Detailed search strategy in PubMed was given in the supplementary material.

### 2.2. Inclusion and Exclusion Criteria

Trails met the following inclusion criteria: (I) type of studies: only randomized controlled trials were included; (II) types of participants: stroke was confirmed by neurological examination or by brain scanning, or both. Patients were not limited by gender and age; (III) types of interventions: the intervention was EA plus SLT; (IV) the comparison was SLT alone; (V) types of outcomes: language functions (oral expression, comprehension, repetition, naming, and reading) and effective rate. Language functions were assessed by scales including western aphasia battery (WAB) [19], China rehabilitation research center aphasia examination (CRRCAE) [20], and aphasia battery of Chinese (ABC) [21]. The definition of the effective rate: effective rate = (“total number of patients” - “number of patients with no response”) /total number of patients, and “no response” meant no significant change in any aspect of language function or regression of one aspect of language function after treatment [22]; and (VI) it was published in English or Chinese language.

The exclusion criteria were as follows: (I) duplicate studies, duplications; (II) full text cannot be obtained through various approaches or studies in which data cannot be extracted; and (III) aphasia caused by other diseases.

### 2.3. Data Extraction and Outcome Measures

For literature selection, two independent reviewers read the titles and abstracts in the first screening stage, read the full texts in the final screening stage, and assessed the articles based on the inclusion and exclusion criteria. Information including the first author, publication year, sample size, patient characteristics, interventions, and outcomes were extracted from the included trails.

### 2.4. Quality Assessment

The risk of bias was independently assessed by two independent reviewers with the Cochrane risk of bias tool from seven domains: (I) randomization process; (II) allocation concealment; (III) blind method; (IV) outcome assessors; (V) missing outcome data processing; (VI) selection of the reported result; and (VII) other bias.

### 2.5. Statistical Analysis

Data analyses were carried out using Review Manager 5.4 software. The pooled effects were the relative risk (RR) and 95% CI for dichotomous outcomes and the standard mean difference (SMD) with 95% CI for continuous outcomes. Heterogeneity between the trails was determined using *I*^*2*^ statistics. Fixed effects model was used if *I*^*2*^ < 50%; otherwise, a random effects model was used (*I*^*2*^ ≥ 50%). Subgroup analyses were performed on the basis of treatment duration. Sensitivity analyses were carried out by removing each study individually to estimate the quality and consistency of the results. Publication bias was carried out with funnel plot.

## 3. Results

### 3.1. Literature Search

**Figure 1:**
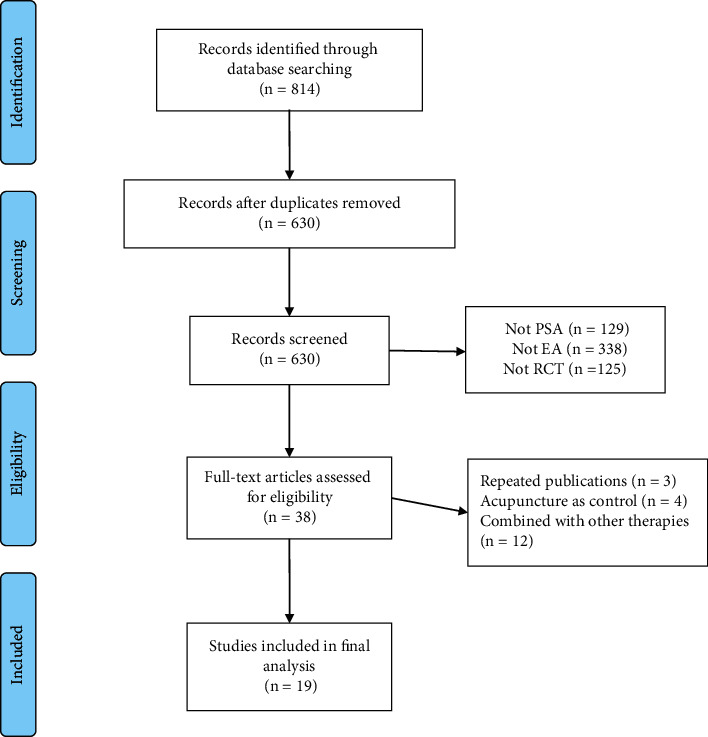
PRISMA flowchart for literature selection.

A total of 814 records were obtained from the eight databases and 184 duplicates were excluded. 630 records were removed after the titles and abstracts were screened. Eventually, 38 records were identified for full-text analysis, and 19 trails [23–41] were deemed eligible finally (Figure 1).

### 3.2. Characteristics of Included Studies

The included trails with sample sizes ranged from 20 to 120 published between 2000 and 2021. In total, 1263 subjects were included, with 638 in EA groups and 625 in control groups. The treatment cycle lasted 10 to 40 days, and each treatment lasted 15–60 min. More details are shown in Table 1.

### 3.3. Study Quality

**Figure 2:**
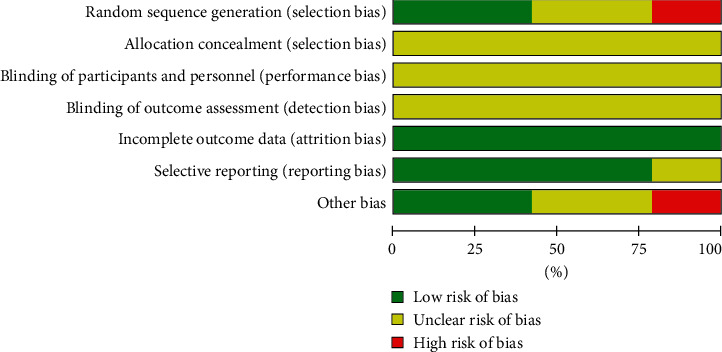
Risk of bias percentage chart.

**Figure 3:**
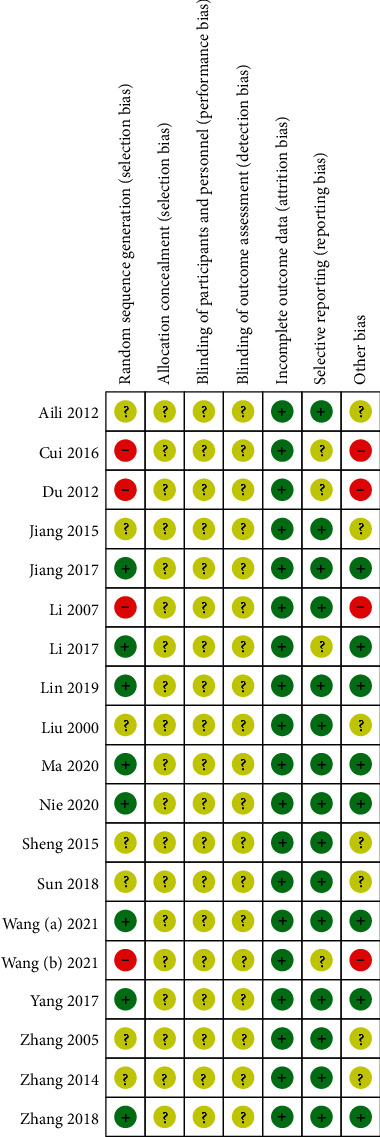
Risk of bias distribution diagram.

A summary of the risk of biases is presented in Figures 2 and 3. With regards to random sequence generation, four studies [24, 33, 37, 39] had a high risk of bias. To reduce the impact of high risk of bias on the pooled results, these four trails [24, 33, 37, 39] were excluded from the performed meta-analysis. With regards to allocation concealment and blinding, all studies had an unclear risk of bias. All trails had a low risk of bias in incomplete outcome data. With regards to other sources of bias, eight studies had a low risk of bias.

### 3.4. Meta-Analysis

#### 3.4.1. Effective Rate

**Figure 4:**
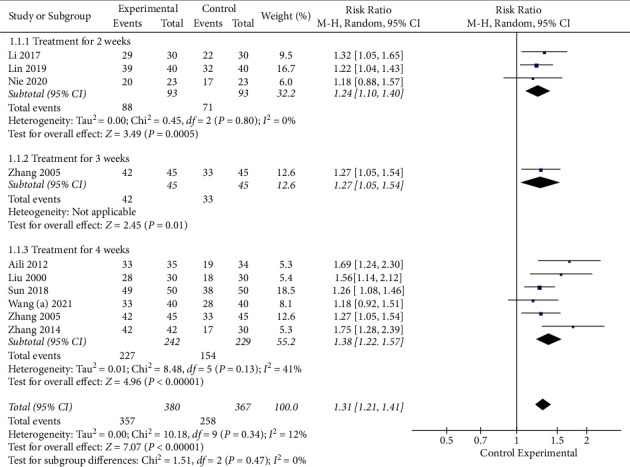
Meta-analysis in effective rate.

**Figure 5:**
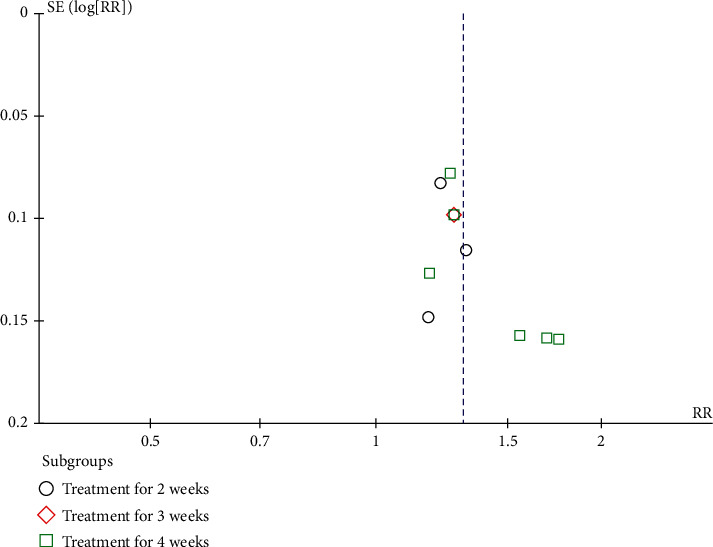
A funnel plot of effective rate.

11 studies with a total of 747 subjects used the effective rate to evaluate the efficacy. A random-effect model was applied due to huge clinical heterogeneity in RCT, like acupoints and manipulation. The pooled analysis showed that EA combined with SLT had a higher effective rate (RR 1.31, 95% CI [1.21, 1.41]). In the subgroup analyses based on treatment duration, both subgroups showed statistically significant improvements in the effective rate with combined treatment compared to SLT alone (treatment for 2 weeks: RR 1.24, 95% CI [1.10, 1.40]; treatment for 3 weeks: RR 1.27, 95% CI [1.05, 1.54]; treatment for 4 weeks: RR 1.38, 95% CI [1.22, 1.57]). More details are shown in Figure 4,. The sensitivity analysis performed by the exclusion method showed that the study by Yang et al. [30] was the main cause of heterogeneity. In addition, the funnel plot was not symmetrical (Figure 5), which did not mean that there was a risk of publication bias because the sample size in this study was not small.

#### 3.4.2. Oral Expression Score

**Figure 6:**
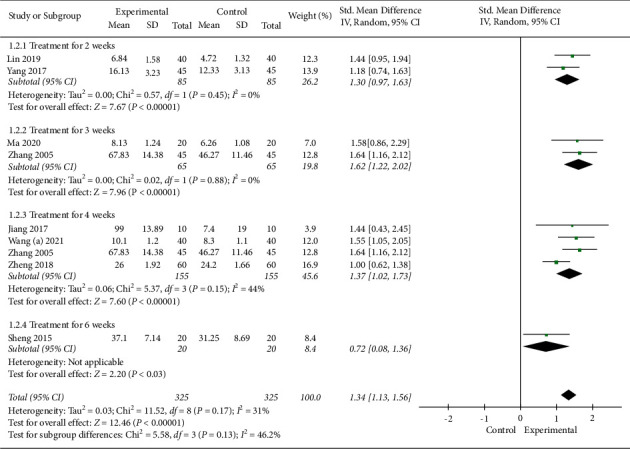
Meta-analysis in oral expression score.

Nine studies with a total of 650 subjects used the oral expression score to evaluate the efficacy. A random-effect model was applied, the pooled analysis showed that EA combined with SLT had a higher oral expression score (SMD 1.34, 95% CI [1.13, 1.56]). In the subgroup analysis based on treatment duration, both subgroups showed statistically significant improvements in oral expression score with combined treatment compared to SLT alone (treatment for 2 weeks: SMD 1.30, 95% CI [0.97, 1.63]; treatment for 3 weeks: SMD 1.62, 95% CI [1.22, 2.02]; treatment for 4 weeks: SMD 1.37, 95% CI [1.02, 1.73]; and treatment for 6 weeks: SMD 0.72, 95% CI [0.08, 1.36]). More details are shown in Figure 6.

The sensitivity analysis performed by the exclusion method showed that the study by Nie et al. [25] was the main cause of heterogeneity.

#### 3.4.3. Comprehension Score

**Figure 7:**
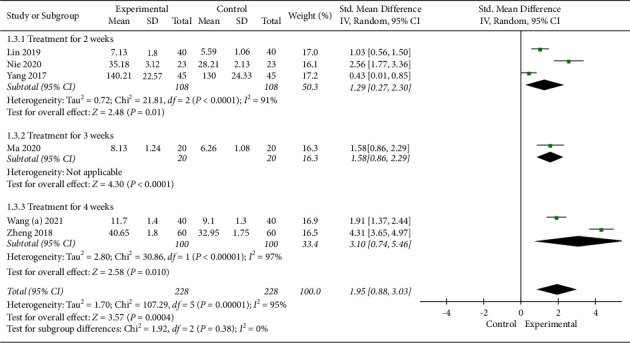
Meta-analysis in comprehension score.

Six studies with a total of 456 subjects used the comprehension score to evaluate the efficacy. A random-effect model was applied, the pooled analysis showed that EA combined with SLT had a higher comprehension score (SMD 1.95, 95% CI [0.88, 3.03]). In the subgroup analysis based on treatment duration, both subgroups showed statistically significant improvements in comprehension score with combined treatment compared to SLT alone (treatment for 2 weeks: SMD 1.29, 95% CI [0.27, 2.30]; treatment for 3 weeks: SMD 1.58, 95% CI [0.86, 2.29]; and treatment for 4 weeks: SMD 3.10, 95% CI [0.74, 5.46]). More details are shown in Figure 7.

The sensitivity analysis performed by the exclusion method showed that no significant changes in heterogeneity were observed.

#### 3.4.4. Reading Score

**Figure 8:**
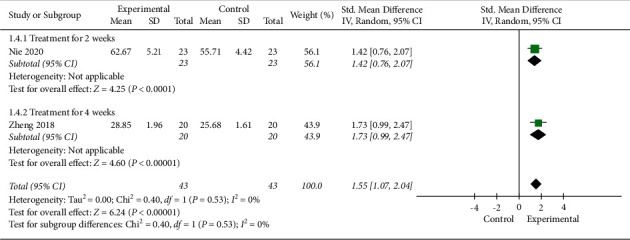
Meta-analysis in reading score.

Two studies with 86 subjects used the reading score to evaluate the efficacy. A random-effect model was applied, the pooled analysis showed that EA combined with SLT had a higher reading score (SMD 1.55, 95% CI [1.07, 2.04]). In the subgroup analysis based on treatment duration, both subgroups showed statistically significant improvements in reading score with combined treatment compared to SLT alone (treatment for 2 weeks: SMD 1.42, 95% CI [0.76, 2.07] and treatment for 4 weeks: SMD 1.73, 95% CI [0.99, 2.47]). More details are shown in Figure 8.

The sensitivity analysis performed by the exclusion method showed that no significant changes in heterogeneity were observed.

#### 3.4.5. Repetition Score

**Figure 9:**
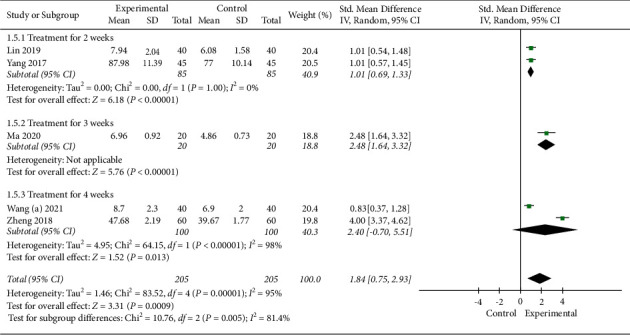
Meta-analysis in repetition score.

Five studies with 410 subjects used the repetition score to evaluate the efficacy. A random-effect model was applied, the pooled analysis showed that EA combined with SLT had a higher repetition score (SMD 1.84, 95% CI [0.75, 2.93]). In the subgroup analysis based on treatment duration, subgroups showed statistically significant improvements in repetition score with combined treatment compared to SLT alone (treatment for 2 weeks: SMD 1.01, 95% CI [0.69, 1.33] and treatment for 3 weeks: SMD 2.48, 95% CI [1.64, 3.32]), however, with no evidence of benefit from treatment for 4 weeks (SMD 2.40, 95% CI [−0.70, 5.51]). More details are shown in Figure 9.

The sensitivity analysis performed by the exclusion method showed that no significant changes in heterogeneity were observed.

#### 3.4.6. Naming Score

**Figure 10:**
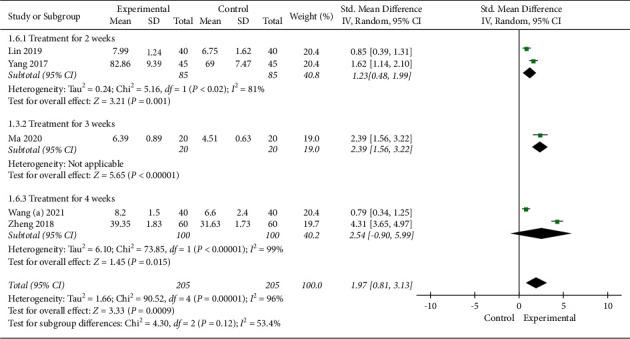
Meta-analysis in naming score.

Five studies with 410 subjects used the naming score to evaluate the efficacy. A random-effect model was applied, the pooled analysis showed that EA combined with SLT had a higher naming score (SMD 1.97, 95% CI [0.81, 3.13]). In the subgroup analyses based on treatment duration, subgroups showed statistically significant improvements in naming score with combined treatment compared to SLT alone (treatment for 2 weeks: SMD 1.23, 95% CI [0.48, 1.99] and treatment for 3 weeks: SMD 2.39, 95% CI [1.56, 3.22]), however, with no evidence of benefit from treatment for 4 weeks (SMD 2.54, 95% CI [-0.90, 5.99]). More details are shown in Figure 10.

The sensitivity analysis performed by the exclusion method showed that no significant changes in heterogeneity were observed.

## 4. Discussion

Aphasia is a common complication following a stroke, often interfering with everyday activities, social abilities, and rehabilitation. In China, acupuncture has a long history of treating PSA, and its efficacy has been supported by evidence-based medical evidence [22]. As an extended technique of acupuncture, EA has both the effects of traditional acupuncture and the functions of modern electrotherapy and is widely used as a complementary therapy for poststroke rehabilitation. An increasing number of RCTs have begun to investigate the effects of EA in patients with PSA. However, there is no uniform conclusion on whether the combination of EA and SLT has positive clinical efficacy in PSA. To systematically collate, appraise, and synthesize the evidence, we conducted this meta-analysis of RCTs.

### 4.1. Summary of Main Findings

Comprehensive analysis of this meta-analysis revealed that subjects treated using combined EA and SLT showed significant improvements in effective rate, oral expression score, comprehension score, repetition score, naming score, and reading score compared to those treated by SLT alone. Therefore, we tentatively conclude that EA combined with SLT as an adjunctive for PSA can increase its clinical effectiveness. However, this conclusion must be considered with cautious, given there was too little information in most of these included trails. Firstly, the processes of randomization, allocation concealment, and binding of most trails are unclear, which may have led to a high risk of bias. Secondly, none of the included RCTs applied statistical methods to estimate the sample size, which resulted in the small sample size included in the study and therefore lowering the credibility of the evidence. In addition, all included studies assessed outcomes before and immediately after EA treatment, while the treatment duration was 10–40 days; therefore, this present study failed to further assess the long-term effects of EA on PSA. Moreover, the implementation program of EA was not uniform and showed large differences in acupoint selection, stimulation methods, needle retention time, and treatment period and frequency, which might have increased the source of heterogeneity [14]. Furthermore, all of the included trails were conducted in China, which may have led to publication bias.

### 4.2. Agreements and Disagreements with Other Published Reviews

Previous systematic reviews/meta-analyses [7–13] have almost all revealed the benefits of acupuncture on PSA. Our review agrees with other studies in the aspect that EA as an adjunct therapy on PSA, though with uncertainty. As an extended technique of acupuncture, studies on systematic synthesis of the evidence on EA for PSA are relatively lacking. A network meta-analysis [15] concluded that the efficacy of EA combined with SLT for PSA was superior to SLT alone in effective rate. The results of this meta-analysis in effective rate are consistent with this network meta-analysis [15]. Furthermore, we also performed subgroup analyses based on treatment duration and assessed the effect of EA on oral expression score, comprehension score, repetition score, naming score, and reading score. In addition, a systematic review [16] of 10 RCTs involving 756 patients conducted in Korea concluded that EA could be considered as an adjunctive therapy for PSA. The difference with our meta-analysis was that it did not perform a quantitative synthesis to assess the relative effect of EA on PSA. Our pooled results are more conducive to the certainty of definitive evidence.

### 4.3. Implications for Research

Of the 19 included trials, only 8 was rated as low risk bias in randomization process, and none of which reported allocation concealment and blinding information. The sample sizes of the studies ranged from 20 to 120, studies with larger sample sizes, clear information about randomization and allocation concealment methods, and statements about whether participants, personnel, and outcome assessors were blinded are needed to assess the effectiveness of EA for PSA. Future studies should pay particular attention to the effects of EA on long-term functional outcomes. It is worth noting that the EA protocols in each study were diverse, including point selection and stimulation duration; therefore, a more standardized and uniform EA treatment protocol should be advocated, which would also facilitate the promotion of EA. In addition, the studies were all conducted in China, and further reliable studies in other ethnic populations are needed to determine population-specific response differences.

### 4.4. Potential Mechanism of Action

Although there is currently limited evidence of EA for PSA, the mechanism by which EA improve symptoms of PSA is being confirmed. An MRI study [42] revealed that the language-related brain areas can be activated through EA treatment. A wide range of brain functional areas such as frontal lobe, occipital lobe, parietal lobe, temporal lobe, precuneus, and insula showed active hyperintensity after EA treatment [42]. Similarly, another MRI study also confirmed this finding [43], that stimulation of acupoints associated with language deficits can selectively activate the brain on the lesional side of PSA patients. In addition, it has been found that EA helps to increase blood perfusion in higher speech centers, which in turn improves the ischemic and hypoxic state of brain tissue and awakens nerve cells [44]. The clinical findings were also demonstrated in rat experiments [45]. After receiving EA intervention, the researchers observed significant proliferation of endogenous neural stem cells in rats with cerebral ischemia-reperfusion injury, suggesting that EA can promote the repair of neurological function and reduce secondary nerve injury [45]. Hence, from the potential mechanism of action, EA seems to be a promising method for the treatment of PSA.

### 4.5. Limitations

There were several potential limitations in this meta-analysis. Firstly, because the included trials lacked follow-up information on EA for PSA, this study could not provide long-term effects of EA for PSA. Secondly, although different acupoint combinations have a significant effect on PSA, our meta-analysis only focused on the overall clinical effect of EA in the treatment of PSA, but did not evaluate the acupoint combination, there it could not provide a basis for specific acupoint selection strategies [46, 47]. Furthermore, the great differences in acupoints pose a challenge to the quantitative findings of this study, so future RCTs should be advocated to adopt standard EA treatment protocols and reduce the generation of heterogeneity to produce more persuasive results.

## 5. Conclusion

The modality of EA combined with SLT for PSA may improve clinical effectiveness compared to SLT alone, which provides a new option for clinical decision-making. However, limited data, poor methodological quality, and potentially exaggerated effect size evaluation limit the quality of the evidence. More high quality, multi-centers RCTs with large samples are still needed to provide higher evidence.

## Figures and Tables

**Table 1:** Descriptive analysis of the characteristics.

First author; year	Simple		Age		Time post onset		Acupoints	Duration & frequency of trial period	Main outcomes
	I	C	I	C	I	C			
Wang et al. [23]. 2021	40	40	52.25 ± 4.71	52.30 ± 4.76	27.32 ± 3.83d	29.21 ± 3.85	DU16 (风府), DU15 (哑门)	20 min each time, once daily, 4 w	Oral expression score, comprehension score, repetition score, naming score, and ER
Wang [24]. 2021	25	25	Unclear	Unclear	Unclear	Unclear	DU20 (百会), Ex-HN13(金津), HT5 (通里), RN23(廉泉)	30 min each time, once daily, 4 w	Oral expression score, comprehension score, repetition score, naming score, and ER
Nie et al. [25]. 2020	23	23	51.0 ± 2.31	52.0 ± 3.12	Unclear	Unclear	Scalppoints	15 min each time, once daily, 10 d	Oral expression score, reading score, comprehension score, and ER
Ma et al. [26]. 2020	20	20	52.15 ± 9.82	51.36 ± 10.11	60.87 ± 21.43d	59.18 ± 24.21d	Scalppoints	30 min each time, once daily, 3 w	Oral expression score, comprehension score, repetition score, and naming score
Lin et al. [27]. 2019	40	40	52.25 ± 4.71	52.30 ± 4.76	Unclear	Unclear	MS6 (顶颞前斜线), MS10 (颞前线), EX-HN1(四神聪), DU16 (风府), DU20 (百会), EX-HN3(印堂), PC6(内关), DU26(水沟)、HT5(通里)、SP6(三阴, RN23(廉泉)	30 min each time, once daily, 2 w	Oral expression score, comprehension score, repetition score, naming score, and ER
Zheng et al. [28]. 2018	60	60	53.58 ± 1.81	58.38 ± 1.31	69.23 ± 4.32d	79.15 ± 3.53d	RN23(廉泉), GB8 (率谷)	30 min each time, once daily, 4 w	Oral expression score, comprehension score, reading score, repetition score, and naming score
Sun [29]. 2018	50	50	53.7 ± 5.2	52.3 ± 4.9	42.1 ± 12.5d	41.3 ± 11.2d	EX-HN13(玉液), Ex-HN13(金津)	20 min each time, once daily, 4 w	ER
Yang et al. [30]. 2017	45	45	58.4 ± 10.38	60.6 ± 11.57	8.68 ± 3.24d	6.78 ± 3.25d	Scalppoints, DU16 (风府), DU15 (哑门), DU20 (百会)	20 min each time, 5 times weekly, 2 w	Oral expression score, comprehension score, repetition score, naming score, and ER
Li et al. [31]. 2017	30	30	57.10 ± 10.03	58.11 ± 9.96	21.33 ± 5.16 d	22.10 ± 4.89 d	MS6 (顶颞前斜线), MS10 (颞前线), EX-HN1(四神聪), DU20 (百会), RN23(廉泉)	30 min each time, twice daily, 14d	Oral expression score, comprehension score, repetition score, naming score, and ER
Jiang et al. [32]. 2017	10	10	63.7 ± 6.6	58.7 ± 10.4	90.1 ± 58.2d	69.6 ± 43. 5d	MS6 (顶颞前斜线), MS7(顶颞后斜线), DU20 (百会)	60 min each time, twice daily, 2w	Oral expression score and ER
Cui et al. [33]. 2016	33	33	56.1 ± 11.0	56.3 ± 10.7	42.2 ± 19.3d	42.1 ± 19.5d	EX-HN13 (玉液), Ex-HN13 (金津)	20 min each time, once daily, 4w	Repetition score, naming score, and ER
Sheng et al. [34]. 2015	20	20	55.05 ± 9.27	57.10 ± 8.30	24.20 ± 10.95d	23.00 ± 10.40d	Unclear	30 min each time, twice daily, 4w	Oral expression score
Jiang et al. [35]. 2015	30	30	57 ± 10	57 ± 9	42.3 ± 19.2d	40.3 ± 19.4d	Unclear	30 min each time, twice daily, 4w	Repetition score and ER
Zhang et al. [36]. 2014	42	30	62.4 ± 1.4	57.6 ± 1.6	78.0 ± 8.6d	85.0 ± 9.2d	EX-HN13 (玉液), Ex-HN13 (金津)	20 min each time, once daily, 1m	ER
Du [37]. 2012	30	30	42∼74	44∼74	7∼67d	9∼66d	RN23 (廉泉)	30 min each time, twice daily, 4w	Oral expression score and ER
Aili et al. [38]. 2012	35	34	Unclear	Unclear	Unclear	Unclear	HT5 (通里), ST36 (足三里), KI6 (照海), PC6 (内关), LI4 (合谷), ST40 (丰隆),	30 min each time, once daily, 1m	ER
LI et al. [39]. 2007	30	30	54 ± 7.8	53 ± 5.6	7 ± 3.2d	7 ± 3.3d	RN23 (廉泉), EX-HN13 (玉液), Ex-HN13 (金津), EX-HN13 (翳明), GB20 (风池)	30 min each time, once daily, 20 d	ER
Zhang et al. [40]. 2005	45	45	56.7 ± 15.6	58.4 ± 13.3	24.4 ± 20.1d	23.4 ± 20.3d	RN23 (廉泉)	30 min each time, once daily, 3w	Oral expression score and ER
Liu et al. [41]. 2000	30	30	Unclear	Unclear	3w-6m	3w-6m	GB8 (率谷), GB13 (本神), DU20 (百会), GB20 (风池)	30 min each time, once daily, 30 d	Oral expression score and ER

## Data Availability

The datasets used in the present review are available from the corresponding author on reasonable request.

## Abbreviations

PSA: Poststroke aphasia
SLT: Speech and language therapy
RCTs: Randomized clinical trials
ER: Effective rate.

## Supplementary Materials

Supplementary Materials Supplementary material: detailed search strategy for PubMed.
