# Supplementary material for: The Effects of Electroacupuncture as an Adjunct Therapy on Poststroke Aphasia: A Systematic Review and Meta-Analysis
Source: Evid Based Complement Alternat Med. 2022 Aug 5;2022:1271205. doi: 10.1155/2022/1271205 (PMC9374558; doi:10.1155/2022/1271205)
Supplement: Supplementary Materials — Supplementary material: detailed search strategy for PubMed. [file 1271205.f1.docx]

**Additional file:** The search strategy for PubMed

*Pubmed:*

#1 Cerebral Hemorrhage[Mesh] OR Brain Infarction[Mesh] OR Stroke[Mesh] OR Cerebrovascular Disorders[Mesh]

#2 Cerebrovascular Disorder*[Title/Abstract] OR Stroke*[Title/Abstract] OR Brain Infarction*[Title/Abstract] OR Cerebral Hemorrhage*[Title/Abstract] OR Intracranial Vascular Disease*[Title/Abstract] OR Cerebrovascular Disease*[Title/Abstract] OR Brain Vascular Disorder*[Title/Abstract] OR Cerebrovascular Occlusion*[Title/Abstract] OR Cerebrovascular Insufficiency*[Title/Abstract] OR Cerebrovascular Accident*[Title/Abstract] OR Cerebrovascular Apoplexy[Title/Abstract] OR Brain Vascular Accident*[Title/Abstract]OR Apoplexy[Title/Abstract] OR Anterior Cerebral Circulation Infarction[Title/Abstract] OR Cerebrum Hemorrhage*[Title/Abstract] OR Intracerebral Hemorrhage*[Title/Abstract] OR brain hemorrhage[Title/Abstract]

#3 #1 OR #2

#4 Aphasia[Mesh] OR Language Disorders[Mesh] OR Speech Disorders[Mesh] OR Speech-Language Pathology[Mesh] OR Rehabilitation of Speech and Language Disorders[Mesh]

#5 Aphasia[Title/Abstract] OR Language Disorders[Title/Abstract] OR Speech Disorders[Title/Abstract] OR Speech-Language Pathology[Title/Abstract] OR Rehabilitation of Speech and Language Disorders[Title/Abstract] OR Alogia*[Title/Abstract] OR Dysphasia[Title/Abstract] OR Dyslalia[Title/Abstract]

#6 #4 OR #5

#7 Acupuncture[Mesh] OR Acupuncture Therapy[Mesh]

#8 Acupuncture[Title/Abstract] OR acupuncture therapy[Title/Abstract] OR electroacupuncture[Title/Abstract] OR electroaucpuncture therapy[Title/Abstract] OR electric acupuncture[Title/Abstract] OR electrical acupuncture[Title/Abstract] OR electrical stimulation therapy[Title/Abstract]

#9 #7 OR #8

#10 Randomized Controlled Trials as Topic[Mesh]

#11 Randomized Controlled Trials[Title/Abstract]OR random*[Title/Abstract] OR controlled clinical trial[Title/Abstract] OR rct[Title/Abstract]

#12 #10 OR #11

#13 #3 AND #6 AND #9 AND #12
